# Maternal Programming of Nursery Pig Performance and Gut Microbiome through Live Yeast Supplementation

**DOI:** 10.3390/ani14060910

**Published:** 2024-03-15

**Authors:** Kayla Law, Lee J. Johnston, Pedro E. Urriola, Andres Gomez

**Affiliations:** 1 Department of Animal Science, University of Minnesota, St. Paul, MN 55108, USA; steve930@umn.edu (K.L.); johnstlj@umn.edu (L.J.J.); urrio001@umn.edu (P.E.U.); 2 West Central Research and Outreach Center, University of Minnesota, Morris, MN 56267, USA

**Keywords:** live yeast, maternal programming, microbiome, piglet, sow

## Abstract

**Simple Summary:**

Post-weaning diarrhea is a critical health and welfare issue in swine production systems as the transition from sow milk to solid feed is a stressful event that is accompanied by microbiome community disturbances that can require antimicrobial treatment or other individualized dietary interventions. This study investigated how feeding sows live yeast during gestation and lactation affects the growth and microbiomes of their offspring in the post-weaning period, without directly feeding live yeast to offspring. The offspring of yeast-fed sows displayed altered growth performance and gut microbiome composition when compared to offspring born to sows fed a control diet. The microbiomes of offspring born to yeast-fed sows contained more taxa associated with beneficial fermentative processes, indicating that the manipulation of offspring microbiomes through maternal diet is a viable method for conferring beneficial microbiome characteristics to offspring in swine systems.

**Abstract:**

The supplementation of live yeast in pig diets is common in the post-weaning phase due to its prebiotic and probiotic effects, but little is known regarding the potential of feeding live yeast to gestating or lactating sows for transferring such benefits to their offspring through maternal programming. The objective of this study was to investigate the effects of live yeast supplementation in sow diets during late gestation and lactation on their reproductive performance and its impact on offspring performance and gut microbiomes in the post-weaning period. Three dietary treatments were imposed on 92 mixed-parity sows during late gestation and lactation based upon the inclusion level of live yeast in corn/soybean meal-based diets: Control (0% yeast), Low (0.1% yeast), and High (0.5% yeast). Nursery pigs in the Low group displayed the highest feed intake in the post-weaning period and greater total gain and average daily gain in comparison to pigs in the High group. The gut microbiomes of nursery pigs differed in composition according to maternal dietary treatment groups at days 4 and 28 post weaning, highlighting higher abundances of bacterial genera typically associated with fermentation roles in the gut microbiomes of offspring of yeast-fed sows. These results indicate that the supplementation of live yeast in sow diets, depending on the inclusion level, may result in beneficial performance and specific microbiome traits for their offspring in the post-weaning period.

## 1. Introduction

Maternal programming refers to the process by which maternal characteristics can predetermine or influence the growth and development of offspring. In swine production systems, several maternal factors have been associated previously with the growth performance, health status, and microbiome composition of offspring [1,2,3,4]. Due to the intrinsic ties between gut microbial communities and host health, maternal programming strategies that aim to target gut microbiome composition in their offspring represent a novel approach to favorably influence their growth and development. One of the most compelling factors influencing piglet performance and microbiomes in early life is maternal diet. Maternal diets during gestation and lactation can affect the initial seeding of piglet gut microbiomes early in life, potentially impacting their physiological performance.

Previous researchers demonstrated that the manipulation of maternal diets altered the composition of gut microbiomes in suckling pigs [5,6,7,8]. However, whether these changes persist past weaning into the nursery phase of production is unclear. The gut microbiome of newborn piglets diversifies quickly in the pre-weaning period, with increasing microbial diversity throughout each life phase in swine production systems [9,10,11,12]. A distinct shift in gut microbiome composition occurs at weaning in tandem with the shift from dietary oligosaccharides found in sow milk to more complex carbohydrates found in plant-based solid feed [1,13,14]. This shift is also accompanied by increased susceptibility to health challenges and post-weaning diarrhea (PWD) in the weeks immediately after weaning, which can decrease growth performance in mild cases and cause death in severe cases [15,16]. Consequently, the weaning and nursery phases of swine production are often the target of investigations involving dietary feed additives to prevent or ameliorate these challenges [17].

Supplementation with live yeast has been studied extensively in food animal nutrition due to its prebiotic and probiotic potential. Previous research reported the effects of live yeast supplementation on microbiome composition and favorable immune function in food animals [18,19,20]. Live yeast can act as a probiotic through various potential mechanisms including the competitive exclusion of pathogen growth, the production of antimicrobial peptides, and the activation of host immune pathways [18]. Beneficial prebiotic effects associated with live yeast are attributable to mannan oligosaccharides and β-glucans found in yeast cell walls [19,21,22] that can stimulate beneficial bacteria, eliciting a wide variety of immunogenic effects and resulting in increased growth performance [22,23,24,25,26]. The supplementation of diets for suckling and nursery pigs with live yeast supported increased average daily gain (ADG) and improved fecal consistency [27,28,29]. However, potential effects on post-weaning pig performance by supplementing only sow diets with live yeast have not been reported. Therefore, the objective of this study was to investigate the effects of feeding live yeast to gestating and lactating sows on their reproductive performance and growth performance of their offspring in lactation and throughout the nursery period. In addition, we studied the effects of maternal yeast feeding on the gut microbiome of weaned offspring.

## 2. Materials and Methods

### 2.1. Animals, Housing, and Diets

Mixed-parity, crossbred sows (*n* = 92; Topigs NorsvinTN 70 females) from three contemporary farrowing groups were used in this experiment. Sows were assigned based on parity to one of three dietary treatments which included: 0 (Control, C), 0.1% (Low, L) or 0.5% (High, H) of a live yeast additive (*Saccharomyces cerevisiae* Sc47, Lesaffre Yeast Corporation, Milwaukee, WI, USA). The diets were mixed using pre-blends of live yeast to ensure a uniform distribution of the yeast product throughout the final diets. Pre-blends for the Control group did not include the yeast feed additive. Dietary treatments were imposed from approximately day 85 of gestation (Table 1) and continued through lactation (Table 2) until piglets were weaned (at about 19 days old). Sows were fed their assigned gestation diet (1.8 kg/head/day) from day 85 to day 110 of gestation on an as-fed basis. At day 111 of gestation, sows were fed their assigned lactation diet (3.6 kg/head/day) until parturition. Sows were allowed ad libitum access to their assigned lactation diet beginning the first day postpartum until piglets were weaned, with diets formulated to meet or exceed nutrient recommendations set by the National Research Council [30] for gestating and lactating sows. Metabolizable energy (ME) was calculated from the ME density of the ingredients [30].

Sows were split into 3 contemporary farrowing groups containing about 30 sows each, with dietary treatment groups assigned randomly within each farrowing group. Across all 3 farrowing groups, treatments were balanced for sow parity. The sows were moved from group pens in a straw-bedded hoop gestation barn on day 78 of gestation to individual stalls with slatted floors in a conventional confinement farrowing barn. Litter size, piglets born live per litter, and stillborn piglets were recorded at birth for all sows prior to cross-fostering. Sow body weight, backfat depth at the last rib, and body condition were recorded on day 85 of gestation, the day prior to the expected farrowing date, and on the day before weaning. Body condition was scored using a caliper device at the last rib as previously described [31]. Parity, gestation length, and lactation length were recorded for all sows. Cross-fostering was allowed only within dietary treatment groups, excluding focal sows that were selected for further microbiome and immune analyses. Piglets born to focal sows were not cross-fostered. Piglets were processed and weighed within 24 h of birth. Piglet processing included tail docking, iron injections, clipping needle teeth, and the castration of males. Piglets were weighed individually within 24 h of birth and the day before weaning.

Pigs from the third farrowing group (*n* = 240; 80 per maternal dietary treatment) were selected randomly for further performance data collection throughout the nursery period. The pigs were balanced by litter, body weight, and sex during nursery allotment. At weaning, the pigs were moved to a research nursery barn in groups based on the maternal dietary treatment group. The treatment groups were kept separate from each other by maintaining one empty pen between the treatment groups. Pigs were housed in pens according to the maternal dietary treatment (10 pigs/pen; 8 pens per maternal dietary treatment). The pigs were provided 0.28 m^2^ of floor space per pig. Each pen was equipped with a stainless-steel feeder with five feeding spaces, a water cup (Drink-o-Mat, Vittetoe Inc., Keota, IA, USA), and slotted plastic flooring over a manure pit. The pigs were weighed individually at the end of each of the 4 dietary phases (d 4, d 14, d 28, d 42). All nursery pigs were allowed ad libitum access to water and a common four-phase feeding program that was devoid of a yeast feed additive. The phase one diet was a proprietary pelleted diet, with antibiotics excluded (First Feed^®^, VitaPlus Corp., Madison, WI, USA), and was fed from d 0 to d 4. The phase two diet was fed from d 4 to d 14 and was a corn and soybean meal-based proprietary blend (Launch^®^, VitaPlus Corp., Madison, WI, USA). Phases 3 and 4 were corn and soybean meal-based (Table 3) and were fed from days 14 to 28 post weaning and days 28–42 post weaning, respectively.

### 2.2. Microbiome Profiling Methods

Fecal samples were collected from all 240 pigs at d 4 and 28 (*n* = 480) post weaning to coincide with the end of dietary phase 1 and phase 3, respectively, and to profile gut microbiome composition without interference from transitions between dietary phases. Only samples from pigs that did not receive antibiotic treatment or experience adverse health conditions were used for microbiome profiling. In total, 180 samples (60/maternal dietary treatment group) from each time-point (*n* = 360 total) were selected randomly for microbiome analysis.

All fecal samples for microbiome analyses were collected with sterile cotton swabs and sterile collection tubes. Fecal swabs were collected from pigs by inserting the cotton swab tips just inside the rectum. All samples were immediately placed on dry ice after collection and then stored at −80 °C until DNA extraction. DNA was extracted from the swabs using PowerSoil Pro DNA extraction kits (Qiagen, Hilden, Germany) following the manufacturer’s instructions. Negative controls, consisting of a sterile blank cotton swab, were created for each individual extraction kit and set of reagents.

Sequence data were generated through targeting the V4 variable region of the 16S rRNA bacterial gene on the MiSeq sequencing platform using the primers 515F (5′-GTGCCAGCMGCCGCGGTAA-3′) and 806R (5′-GGACTACHVGGGTWTCTAAT-3′) and dual-indexing library preparation [32]. Raw sequence data were processed using a custom bioinformatics pipeline to remove primer sequences and perform quality control and filtering, as previously described [2]. Filtered sequence data were then processed using the QIIME2 pipeline [33] and its DADA2 plugin [34]. The Silva database [35] was used to taxonomically identify amplicon sequence variants (ASVs) and estimate their abundances in each sample.

An analysis of the processed 16S rRNA sequence data was performed using various packages in the R statistical software (version 4.2.2) [36]. Sequence data from negative controls generated for each extraction kit were used to screen for potential contamination using the R microDecon package [37]. ASVs that were identified as contaminants were filtered out of the corresponding sequence datasets, and potential sequencing artifacts were removed using the labdsv package by eliminating ASVs present in fewer than 3 samples and with fewer than 5 reads across all the dataset [38]. Beta diversity and principal coordinate analyses were performed using the vegan package [39]. The ANCOMBC package [40] was used to identify discriminant genera among the dietary treatment groups. Taxa were considered statistically significant discriminators for the High and Low treatments if q < 0.05 after false discovery rate (FDR) adjustments and if they showed log-fold change values in relative abundance of at least (+/−) 0.5 compared to the Control group.

### 2.3. Statistical Analyses

Generalized linear mixed models were built for all performance comparisons using the R lme4 package [41]. Models for sows included treatment and farrowing room as fixed effects and farrowing group, parity, and litter sire as random effects. Models for piglets in the pre-weaning period included maternal treatment, farrowing room, and sex as fixed effects and farrowing group, sire breed of sow, and maternal parity as random effects. Models in the nursery period considered treatment, sex, and time as fixed effects and sire breed of sow, maternal parity, and pen as random effects. A *p*-value of <0.05 was considered a significant result. The Tukey–Kramer post hoc test for multiple comparisons was used to differentiate among treatment means. The standard error was reported as a pooled standard error for each comparison. All statistical analyses for comparisons of alpha and beta diversity were based on nonparametric Kruskal–Wallis tests and permutational multivariate analyses of variance (PERMANOVA).

## 3. Results

### 3.1. Sow and Piglet Performance

Sow parity, gestation length, and lactation length did not differ among the dietary treatment groups (Table 4). Dietary treatment had no effect on the total feed intake or average daily feed intake (ADFI) during lactation. Sow body weight, backfat depth at the last rib, and caliper score were unaffected by yeast supplementation at each sampling time-point (Table 5). Similarly, dietary treatment had no effect on the total piglets born, liveborn piglets, stillborn piglets per litter, or the pre-weaning mortality of piglets (Table 6). The average birth weight, weaning weight, and average daily gain (ADG) did not differ among piglets born to sows fed different dietary treatments (Table 7).

### 3.2. Performance and Gut Microbiome Composition of Nursery Pigs

Seven pigs were removed from the study due to death or adverse health conditions. Due to an outbreak of suspected *Streptococcus suis*, which is endemic in the research swine herd, and observed poor overall pig health throughout the nursery barn around day 16 of the experiment, all pigs received amoxicillin continuously through drinking water from days 16 to 20 of the experiment.

**Figure 1:**
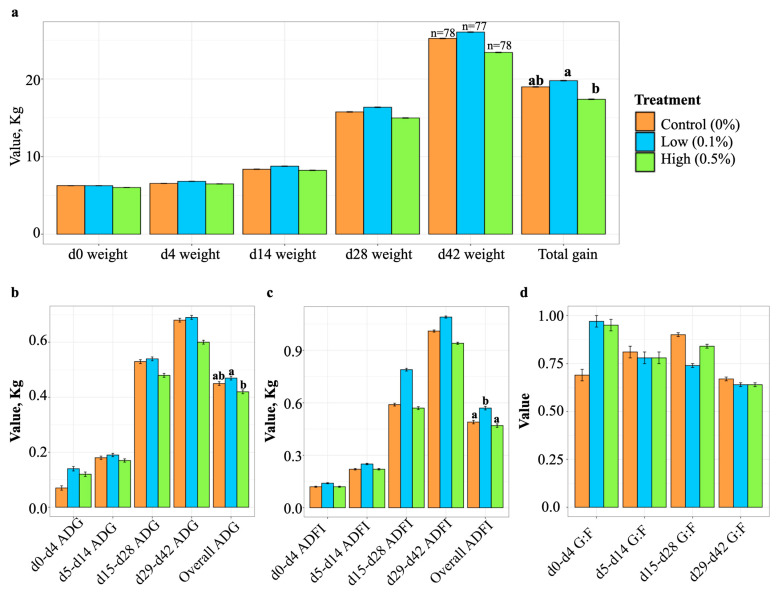
Effects of maternal dietary yeast treatment on nursery pig body weight (**a**), ADG (**b**), ADFI (**c**), and G:F ratios (**d**). ADG was calculated individually, while ADFI was calculated on a pen basis. G:F ratios were calculated by dividing individual ADG by pen ADFI. Error bars represent standard error pooled across treatment groups for each comparison. Differing letter superscripts denote differences (*p* < 0.05) among treatment groups in repeated measures models.

Nursery pigs born to sows fed the high-yeast diet gained less weight throughout the entire nursery period when compared to those born to sows fed the low-yeast diet. Total weight gain did not differ between nursery pigs born to sows in the Control and the Low groups (Figure 1a). Similarly, the overall ADG for the entire nursery period was higher for pigs born to sows fed the low-yeast diet when compared to pigs born to sows fed the high-yeast diet (Figure 1b). However, nursery pigs belonging to the Low treatment group displayed higher ADFI than both the Control or High groups throughout the nursery period (Figure 1c). Gain-to-feed ratios did not differ among the treatment groups (Figure 1d).

**Figure 2:**
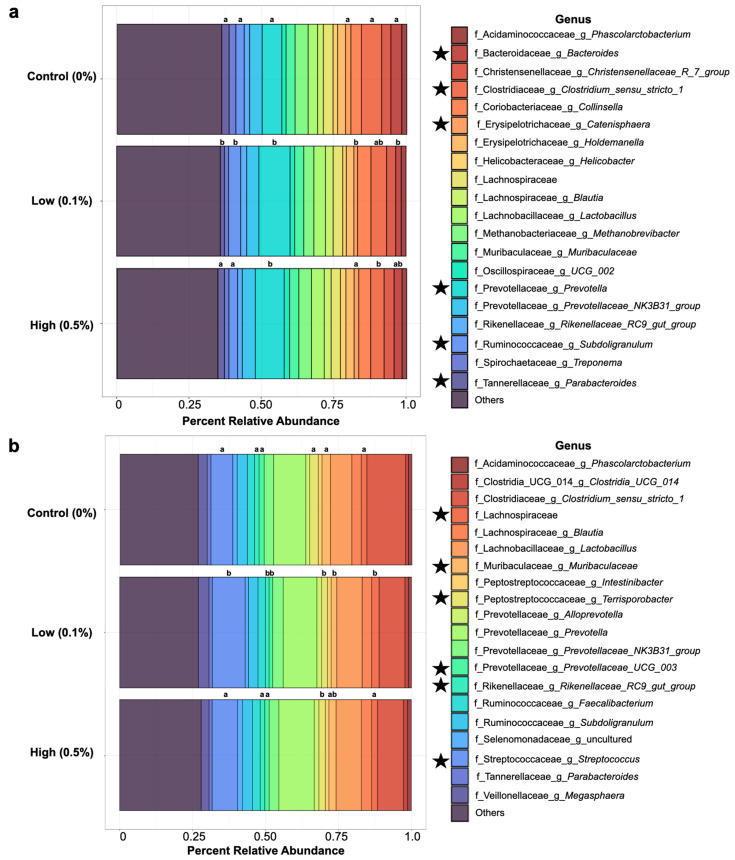
The top 20 most abundant genera in nursery pig gut microbiomes at day 4 post weaning (**a**) and day 28 post weaning (**b**), averaged by maternal dietary treatment. Abundance is expressed as percent relative abundance as a proportion of the total. Listed taxa are identified at the genus level or represent an unidentified genus within the listed family. Star symbols next to the legend represent genera that were identified as different in terms of their abundances among treatment groups. Significance is denoted above each corresponded colored bar by letter superscripts, with differing letters representing differences (*p* < 0.05) in relative abundances.

To visualize and explore potential differences in the microbial community composition of nursery pig gut microbiomes, the top 20 genera in pigs from each treatment group were identified at each sampling time-point and displayed in Figure 2. The relative abundances of each genus were averaged for each maternal treatment group at each time-point. The top 20 genera were consistent across maternal treatment groups at both days 4 (Figure 2a) and 28 (Figure 2b) post weaning. However, differences in the relative abundances of several taxa were observed among the maternal treatment groups (Kruskal–Wallis, *p* < 0.05).

**Figure 3:**
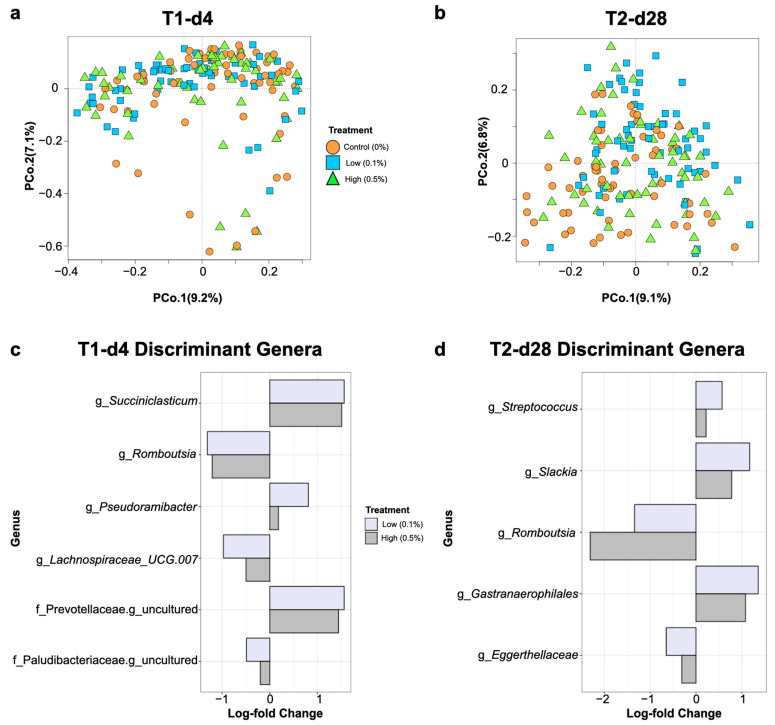
Nursery pig gut microbiome composition at the ASV level at days 4 (**a**) and 28 (**b**) post weaning according to Bray–Curtis dissimilarities. Each shape represents an individual pig, with colors and shapes denoting maternal dietary treatments. Discriminant genera among treatments for nursery pigs were identified by ANCOMBC analysis at each sampling time-point (**c**,**d**). X axis values represent log-fold changes (q < 0.05) in relative abundances of listed genera or an unidentified genus within the listed family. Positive and negative log-fold changes indicate enriched or depleted taxa in low- or high-yeast treatments, respectively, compared with the control.

The overall gut microbiome community composition differed among nursery pigs based on their maternal treatment group at day 4 post weaning (PERMANOVA; *R*^2^ = 0.02, *p*  =  0.001; Figure 3a). Differences in pairwise PERMANOVA comparisons between the control group and the yeast-fed groups for microbiome community composition were also observed, with the largest distinctions observed for the Low group (Control vs. Low: *R*^2^ = 0.02, *p*  =  0.001; Control vs. High: *R*^2^ = 0.01, *p*  =  0.03, Low vs. High: *R*^2^ = 0.02, *p*  =  0.008). At day 28 post weaning, the effect (*R*^2^) of the maternal diet on pig gut microbiome composition increased slightly (PERMANOVA; *R*^2^ = 0.04, *p*  =  0.001; Figure 3b), with significant pairwise differences between the Control and the High/Low groups maintained as well (Control vs. Low: *R*^2^ = 0.05, *p*  =  0.001; Control vs. High: *R*^2^ = 0.02, *p*  =  0.001, Low vs. High: *R*^2^ = 0.03, *p*  =  0.001).

Multivariate ordination of microbial composition appeared to be constrained by unknown factors, particularly at day 4 post weaning as seen in Figure 3a. Hence, the analysis of compositions of microbiomes with bias correction (ANCOMBC) method [40] was implemented for reducing the effects of inherent biases introduced by amplicon sequencing at each time-point. This method revealed that the gut microbiomes of nursery pigs born to yeast-fed sows displayed differential abundances of several genera in comparison to those born to Control sows at days 4 and 28 post weaning, regardless of the yeast inclusion rate. The genera *Romboutsia*, *Lachnospiraceae UCG.007*, and an unidentified genus in the family Paludibacteraceae were identified as discriminant genera for the Control group at day 4 post weaning, while the genera *Pseudoramibacter*, *Succiniclasticum*, and an unidentified genus in the family Prevotellaceae were identified as discriminant genera (q < 0.05) for yeast-fed offspring (Figure 3c). At day 28 post weaning, the gut microbiomes of the offspring of yeast-fed sows were discriminated by higher abundances (q < 0.05) of the genera *Streptococcus*, *Slackia*, and *Gastranaerophilales*, while the offspring of Control sows were discriminated by higher abundances (q < 0.05) of the genus *Romboutsia* and an unidentified genus in the family Eggerthellaceae (Figure 3d).

## 4. Discussion

Here, we show that maternal programming based on the live yeast supplementation of sow diets during late gestation and lactation is associated with differences in growth performance and gut microbiome composition of offspring after weaning. In the post-weaning period, nursery pigs born to sows fed the low (0.1%)-yeast diet displayed greater overall ADFI when compared to pigs in both other maternal dietary treatment groups. Pigs in the Low group also displayed greater body weight gain and ADG in the nursery compared to pigs raised by sows fed the high (0.5%)-yeast diet, suggesting that higher inclusion rates of live yeast are not necessarily more beneficial.

Maternal dietary yeast supplementation did not result in alterations in sow or piglet performance in the pre-weaning period. Similarly, researchers utilizing live yeast products in sow feed in late gestation and lactation observed no effects on sow or piglet performance but did note increased levels of IgG in the colostrum and milk of yeast-fed sows [42,43]. Contrary to these results, other researchers supplementing sow diets with live yeast observed improved sow reproductive performance [44]. The variation in the reported benefits of maternal yeast supplementation in swine systems highlights the need for further research in this area, as well as inherent challenges due to variability among different strains of yeast products.

Previously, researchers demonstrated increased ADG of pigs in the post-weaning period when they were directly fed the same yeast product as used in the study reported herein [28]. The direct supplementation of nursery pigs with the same live yeast product used in our experiment also increased ADG and ADFI in the post-weaning period [45,46]. Similarly, directly feeding other yeast products to nursery pigs increases ADG [29]. Thus, one might easily surmise that feeding live yeast or yeast products directly to nursery pigs would improve growth performance. However, in this experiment, we documented the altered growth performance of nursery pigs through maternal programing, that is, when yeast was included only in maternal diets as opposed to yeast inclusion directly in nursery pig diets. This observation demonstrates an important carryover effect from feeding yeast to dams during gestation and lactation on the post-weaning performance of pigs.

We show that feeding live yeast to sows altered the post-weaning growth performance of their offspring and elicited changes in the gut microbial communities of nursery pigs. The overall structure of the gut microbiome community at days 4 and 28 post weaning differed based upon the maternal dietary treatment, although the magnitude of effects for these comparisons was small. Notably, at day 4 post weaning, pigs born to yeast-fed sows were discriminated by greater abundances of the genus *Pseudoramibacter* which has been associated with the digestion of fermentable carbohydrates and subsequently short-chain fatty acid (SCFA) production [47,48]. The offspring of yeast-fed sows were also discriminated by an unidentified genus in the family, Prevotellaceae, and the genus *Succiniclasticum* both of which harbor SCFA production capabilities [49,50,51]. The gut microbiomes of offspring from yeast-fed sows also harbored greater abundances of the genera *Prevotella* in comparison to the offspring of sows fed the Control diet, which underscores a potential of maternal supplementation with live yeast to impact the fermentative landscape of their offspring gut microbiomes. Specifically, pigs raised by sows fed the low-yeast diet harbored significantly decreased abundances of the genera *Catenisphaera* and *Bacteroides* compared with those raised by the Control sows. High abundances of some taxa from the genus *Bacteroides* in pre-weaning and weaning transition periods have been linked to incidences of post-weaning diarrhea [52]. Increased abundances of these taxa are also associated with the prevalence of enterotoxigenic *Escherichia coli* infections and post-weaning diarrhea [53].

At day 28 post weaning, pigs born to yeast-fed sows were discriminated by greater abundances of the genera *Streptococcus*, *Slackia*, and *Gastranaerophilales*, compared with the offspring of sows fed the control diet. Although the genera identified as discriminant taxa among the maternal treatment groups differed on days 4 and 28 post weaning, observed differences can likely be attributed to the natural diversification of pig gut microbiomes with increasing age [54,55]. Higher abundances of the genus *Slackia* at the end of the nursery phase have previously been positively associated with an increased growth performance [54]. Likewise, the genus *Streptococcus* was significantly more abundant in the gut microbiomes of pigs in the Low yeast group at day 28 post weaning compared to the gut microbiomes of pigs raised by the Control and High sows. Although this genus is composed of commensal species endemic to pig gut microbiomes across life phases [54], it is also composed of species that are potentially pathogenic in swine systems, such as *Streptococcus suis* [56,57]. However, previous research investigating post-weaning diarrhea noted that the proliferation of pathogenic taxa such as *Escherichia coli* was associated with marked reductions in abundances of the genus *Streptococcus* [58,59,60]. Our findings combined with those of previous researchers suggest that feeding yeast to sows may reduce the abundances of potentially pathogenic taxa in the gut microbiomes of their offspring and promote the growth of gut bacteria with potentially beneficial fermentative roles in the post-weaning period, though the exact mechanism is unknown.

## 5. Study Limitations

From these data, we cannot say with certainty that the supplementation of live yeast in sow diets caused the direct transmission of beneficial microbes from sows to piglets or whether observed differences in post-weaning gut microbiome composition were driven by some other mechanism(s). Additionally, the access of suckling piglets to sow feed was limited but not entirely prevented by the design of sow feeders, so piglets may have consumed small amounts of sow feed. The antibiotic treatment of pigs to maintain pig health and welfare between the sampling time-points may have also influenced nursery pig gut microbiome community composition and diversity. While the adverse health event and subsequent administration of oral antibiotic interfered with attempts to ensure antimicrobials did not alter observations of microbial patterns, this intervention mimics a common scenario and management practice in commercial swine productions systems. Future research in this area should profile markers of inflammation and cytokines in piglets in the pre-weaning period, as well as during the weaning transition period, to determine whether maternal live yeast supplementation has immunomodulatory effects that could potentially improve health status in the post-weaning period. Additionally, future studies should attempt to elucidate the mechanism(s) behind the carryover effects of maternal programming observed in this study, which remain unclear.

## 6. Conclusions

The supplementation of sow diets during gestation and lactation with 0.1% of live yeast increased the post-weaning ADFI of offspring. Nursery pigs raised by sows fed 0.1% yeast gained more weight and had greater ADG compared to those raised by sows fed 0.5% yeast. The performance of sows and suckling pigs was not affected by feeding live yeast to sows. Live yeast supplementation in sow diets was associated with greater abundances of several genera linked to fermentation and SCFA production, particularly at day 4 post weaning. These results indicate that maternal programming through live yeast supplementation at an inclusion rate of 0.1% in sow diets is a viable method for promoting the growth of potentially beneficial bacteria in piglet microbiomes that persist into the post-weaning period, though these effects must be balanced with observations of increased feed intake in the post-weaning period.

## Figures and Tables

**Table 1:** Ingredient composition and calculated energy and nutrient content of sow gestation diets (as-fed basis).

Ingredient, %	Control	Low	High
Corn	83.7	83.6	83.2
Soybean meal 46% CP	12.0	12.0	12.0
Mineral/vitamin premix ^a^	4.3	4.3	4.3
Lysine HCl	0.025	0.025	0.025
Active dry yeast	-	0.1	0.5
Total	100.0	100.0	100.0
Calculated energy and nutrient content:			
Metabolizable energy, kcal/kg	3254		
Crude protein, %	11.4		
Fat, %	3.6		
Standardized ileal digestible lysine, %	0.60		
Calcium, %	0.86		
Av. phosphorus, %	0.54		
Salt, %	0.51		

^a^ Contained the following nutrients per kg of premix: Ca, 179 g; av. phosphorus, 87 g; NaCl, 125 g; vitamin A, 275,575 IU; vitamin D, 68,894 IU; vitamin E, 2205 IU; vitamin K, 110 mg; riboflavin, 248 mg; niacin, 1378 mg; pantothenic acid, 827 mg; pyridoxine, 55 mg; vitamin B_12_, 1.38 mg; folic acid, 41 mg; biotin, 8 mg; Zn, 2260 mg; Fe, 1350 mg; Mn, 450 mg; Cr, 5 mg; Cu, 135 mg; I, 55 mg; Se, 7.5 mg.

**Table 2:** Ingredient composition and calculated energy and nutrient content of sow lactation diets (as-fed basis).

Ingredient, %	Control	Low	High
Corn	68.1	68.0	67.6
Soybean meal 46% CP	25.5	25.5	25.5
Mineral/vitamin premix ^a^	4.3	4.3	4.3
Distillers corn oil	2.0	2.0	2.0
Lysine HCl	0.1	0.1	0.1
Active dry yeast	-	0.1	0.5
Total	100.0	100.0	100.0
Calculated energy and nutrient content:			
Metabolizable energy, kcal/kg	3340		
Crude protein, %	16.6		
Fat, %	5.4		
Standardized ileal digestible lysine, %	0.98		
Calcium, %	0.91		
Av. phosphorus, %	0.57		
Salt, %	0.51		

^a^ Contained the following nutrients per kg of premix: Ca, 179 g; av. phosphorus, 87 g; NaCl, 125 g; vitamin A, 275,575 IU; vitamin D, 68,894 IU; vitamin E, 2205 IU; vitamin K, 110 mg; riboflavin, 248 mg; niacin, 1378 mg; pantothenic acid, 827 mg; pyridoxine, 55 mg; vitamin B_12_, 1.38 mg; folic acid, 41 mg; biotin, 8 mg; Zn, 2260 mg; Fe, 1350 mg; Mn, 450 mg; Cr, 5 mg; Cu, 135 mg; I, 55 mg; Se, 7.5 mg.

**Table 3:** Ingredient composition and calculated energy and nutrient content of phase 3 and phase 4 nursery diets.

Ingredient, %	Phase 3 (d 14 to d 28)	Phase 4 (d 28 to d 42)
Corn	53.9	64.24
Soybean meal 46.5% CP	25.0	30.0
Distillers corn oil	1.5	2.0
Whey permeate	9.0	--
Whey, powdered	0.25	--
Hamlet protein 300	3.82	--
Menhaden fish meal	3.25	--
Dicalcium phosphate, 21% P	0.82	0.944
Calcium carbonate	0.52	0.86
White salt	0.40	0.58
L-lysine HCl 98.5%	0.50	0.494
Zinc oxide 72%	0.35	--
Vitamins, trace minerals, amino acids, other	0.67 ^a^	0.884 ^b^
Total	100.0	100.0
Calculated nutrient composition		
Metabolizable energy, kcal/kg	3358.0	3387.5
Crude protein, %	20.63	19.05
Crude fat, %	4.82	5.37
Calcium, %	0.7	0.63
Av. phosphorus, %	0.66	0.55
Standardized ileal digestible (SID) Lysine, %	1.4	1.27

^a^ Contained the following nutrients per kg: total methionine/tryptophan/threonine, 41 g; vitamin A, 60 kIU; vitamin D, 10 kIU; vitamin E, 500 IU; vitamin K, 31 mg; riboflavin, 77 mg; niacin, 355 mg; pantothenic acid, 202 mg; pyridoxine, 27 mg; vitamin B_12_, 0.437 mg; folic acid, 5 mg; biotin, 1.5 mg; Fe, 1220 mg; Mn, 256 mg; Cr, 1 mg; Cu, 104 mg; I, 8.2 mg; Se, 1.6 mg. ^b^ Contained the following nutrients per kg: total methionine/tryptophan/threonine, 112 g; vitamin A, 300 kIU; vitamin D, 54 kIU; vitamin E, 2240 IU; vitamin K, 148 mg; riboflavin, 296 mg; niacin, 1768 mg; pantothenic acid, 1064 mg; pyridoxine, 123 mg; vitamin B_12_, 1.5 mg; folic acid, 15 mg; biotin, 6.8 mg; Zn, 4417 mg; Fe, 7211 mg; Mn, 1308 mg; Cr, 5.4 mg; Cu, 4902 mg; I, 23.8 mg; Se, 8.0 mg.

**Table 4:** Sow parity, gestation and lactation length, and lactation feed consumption for each dietary treatment group.

Observation	Control	Low	High	PSE ^1^
Number of observations	30	31	31	--
Average parity	2.80	2.87	2.94	0.07
Gestation length, d	116.7	116.5	116.5	0.07
Lactation length, d	19.4	19.2	19.5	0.07
Total lactation feed consumption, kg	139.8	140.0	144.1	0.32
Lactation ADFI, kg ^2^	7.26	7.35	7.42	0.07

^1^ Pooled standard error. ^2^ Calculated as total lactation feed intake divided by lactation length for each sow.

**Table 5:** Sow body weight, backfat depth, and caliper scores for each dietary treatment.

Observation	Control ^2^	Low ^2^	High ^2^	PSE ^1^
Body weight, kg:				
Day 85 of gestation	253.7	260.0	254.8	0.35
Day before expected farrowing date	263.2	268.6	264.0	0.34
Day before weaning	240.7	246.1	243.6	0.33
Backfat depth, cm:				
Day 85 of gestation	1.32	1.42	1.41	0.03
Day before expected farrowing date	1.32	1.32	1.31	0.03
Day before weaning	1.17	1.12	1.12	0.03
Caliper score ^3^:				
Day 85 of gestation	16.1	16.4	15.8	0.07
Day before expected farrowing	16.0	16.3	15.5	0.08
Day before weaning	14.5	14.5	14.5	0.07

^1^ Pooled standard error. ^2^ Control (no additional yeast), low (0.1% yeast), and high (0.5% yeast). ^3^ [31].

**Table 6:** Effect of dietary yeast treatments on litter size and piglet mortality.

Observation	Control	Low	High	PSE ^1^
Total pigs born per litter	15.2	17.2	15.7	0.11
Pigs born live per litter	14.2	15.5	14.9	0.11
Stillborn pigs per litter	1.0	1.7	0.9	0.08
Pigs weaned/litter	12.7	12.3	12.1	0.09
Pre-weaning mortality, % ^2^	14	18	17	0.02

^1^ Pooled standard error. ^2^ Calculated as (# of pigs that died in each litter before weaning/litter sizes after cross-fostering) × 100.

**Table 7:** Effect of maternal dietary yeast on piglet body weight and pre-weaning growth rate.

Observation	Control	Low	High	PSE ^1^
Number of observations (birth)	459	533	487	--
Number of observations (weaning)	370	386	380	--
Birth weight, kg	1.43	1.37	1.36	0.002
Weaning weight, kg	6.14	5.92	6.01	0.005
Total weight gain per pig, kg	4.63	4.44	4.59	0.005
ADG, kg ^2^	0.24	0.23	0.24	0.005

^1^ Pooled standard error. ^2^ Piglet average daily gain (ADG) was calculated using each individual sow’s lactation length and litter weight gain.

## Data Availability

The data presented in this study are available on request from the corresponding author. The data are not publicly available due to a temporary embargo held by the first author while their doctoral dissertation is finalized.
